# Contribution of a luminance-dependent S-cone mechanism to non-assimilative color spreading in the watercolor configuration

**DOI:** 10.3389/fnhum.2014.00980

**Published:** 2014-12-09

**Authors:** Eiji Kimura, Mikako Kuroki

**Affiliations:** ^1^Department of Psychology, Faculty of Letters, Chiba UniversityChiba-shi, Japan; ^2^Graduate School of Humanities and Social Sciences, Chiba UniversityChiba-shi, Japan

**Keywords:** watercolor effect, assimilation, color spreading, S cone, visual illusion

## Abstract

In the watercolor configuration composed of wavy double contours, both assimilative and non-assimilative color spreading have been demonstrated depending on the luminance conditions of the inner and outer contours (IC and OC, respectively). This study investigated how the induced color in the watercolor configuration was modulated by combinations of the IC and the OC color, particularly addressing non-assimilative color spreading. In two experiments, the IC color was fixed to a certain color and combined with various colors selected from a hue circle centered at the background white color. Color spreading was quantified with a chromatic cancelation technique. Results showed that both the magnitude and the apparent hue of the color spreading were largely changed with the luminance condition. When the IC contrast (Weber contrast of the IC to the background luminance) was smaller in size than the OC contrast (higher IC luminance condition), the color spreading was assimilative. When the luminance condition was reversed and the IC contrast was greater than the OC contrast (lower IC luminance condition), the color spreading was non-assimilative and yellowish. When the color spreading was analyzed in terms of cone-opponent excitations, the results were consistent with the interpretation that the color spreading is explainable by a combination of chromatic diffusion from the IC and chromatically opponent induction from the OC. The color spreading in the higher IC luminance condition mainly reflected the chromatic diffusion by both (L–M) and S cone-opponent mechanisms. The non-assimilative color spreading in the lower IC luminance condition mostly reflected S-cone mediated opponent induction and the contribution of −S inducing mechanisms was differentially large. These findings provided several constraints on possible visual mechanisms underlying the watercolor effect.

## Introduction

**Figure 1:**
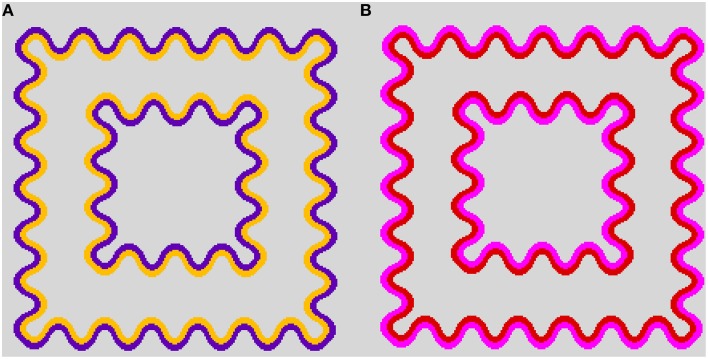
**Demonstrations of color spreading in the watercolor configuration. (A)** Typical assimilative color spreading in a color combination of a light orange inner contour (IC) and a dark purple outer contour (OC). **(B)** Non-assimilative yellow color spreading in a dark red IC and a lighter magenta OC color combination. See also Supplementary Figure 1 in Kimura and Kuroki (2014) for a demonstration of non-assimilative color spreading on different background luminances.

When a wavy dark contour is flanked on the inside by a lighter chromatic contour, the lighter color will spread over the entire enclosed area. This assimilative spreading of the inner contour color is known as the watercolor effect (Figure 1A) (Pinna et al., 2001). Moreover, previous studies have demonstrated that non-assimilative as well as assimilative color spreading occur in the watercolor configuration depending on luminance conditions of the inner contour (IC) and the outer contour (OC) (Pinna and Grossberg, 2005; Pinna, 2006; Kitaoka, 2007; Kimura and Kuroki, 2014). For example, when the IC color is black and the OC color is blue, the induced color in the region delineated by the IC becomes yellow, which is the complementary color of the OC color (Pinna, 2006). Subsequently, Kitaoka (2007) demonstrated non-assimilative yellow spreading in red–magenta (Figure 1B) and green–cyan color combinations.

To elucidate visual mechanisms mediating the watercolor effects, Kimura and Kuroki (2014) investigated luminance conditions suitable for assimilative and non-assimilative color spreading in the watercolor configuration. They found that the luminance condition suitable for non-assimilative color spreading was nearly opposite to that for assimilative color spreading. Assimilative color spreading was strong when the Weber contrast of the IC to the background luminance (IC contrast) was smaller in size (absolute value) than that of the OC (OC contrast) (Pinna et al., 2001; Devinck et al., 2005). Non-assimilative color spreading was strong when the IC contrast was greater than the OC contrast. Moreover, they demonstrated a bilateral color spreading effect, i.e., assimilative color spreading on one side and non-assimilative color spreading on the other side, that resulted from the opposite luminance conditions for assimilative and non-assimilative color spreading. Based on these findings, Kimura and Kuroki (2014) argued that color spreading of two types was mediated by at least partially different visual mechanisms. In addition, the luminance conditions for the watercolor effect have been explored recently (Pinna and Reeves, 2006; Takashima, 2008; Cao et al., 2011; Devinck and Knoblauch, 2012; Coia et al., 2014). In fact, an achromatic watercolor effect has also been found (Takashima, 2008; Cao et al., 2011).

In contrast to the luminance conditions, color conditions for the watercolor effect, particularly for non-assimilative color spreading, have not been explored much. For assimilative color spreading, previous studies demonstrated that numerous color combinations produce the effect when the luminance condition is favorable (Pinna et al., 2001; Devinck et al., 2006a). The magnitude of color spreading varied with color combinations: it was large when a complementary pair of colors was used and became much smaller when the same color was used for the IC and OC colors (Pinna et al., 2001; Devinck et al., 2006a). Devinck et al. (2006a) also showed that color spreading was strong when the IC and the OC were saturated equally, and became weaker when the saturation of one contour was decreased. These results suggest that greater chromatic contrast between the double contours results in stronger color spreading. Moreover, Devinck et al. (2006a) showed that both (L–M) and S-cone opponent mechanisms contribute to assimilative color spreading, but the relative contribution of the (L–M) mechanisms was apparently greater. These results suggest that assimilative color spreading depends on both luminance and color conditions (Devinck et al., 2006a; Kimura and Kuroki, 2014).

For non-assimilative color spreading, Pinna (2006) first reported this atypical color spreading by fixing the IC color to black and combining it with different OC colors of blue, green, yellow, and red. When the observers were asked to report the perceived hue within the region delineated by the double contours, they reported a complementary color of the OC for all color combinations, e.g., the perceived color was greenish for the black-red color combination. It is noteworthy that the color spreading was reported in the region delineated by the *IC*, although the induced color was the complementary color of the *OC*. This finding suggested chromatically opponent induction from the OC. However, subsequent evidence has suggested that the induced color is predominantly yellow. Kitaoka (2007) introduced the atypical color spreading with nice demonstrations as the *paradoxical watercolor effect*, in which the test region “appears to be slightly tinted yellow, though there is no yellow” in the IC and OC colors. Recently, Kimura and Kuroki (2014) also showed in cancelation experiments that the color spreading found in similar conditions was mostly yellow rather than complementary to the OC color, although the color combinations examined in their study were limited to orange–purple and red–magenta combinations. Because of these previous findings suggesting that simple chromatically opponent induction might not account for the atypical color spreading, we designate the color spreading as “non-assimilative” in this paper rather than the contrast effect.

Kimura and Kuroki (2014) also reported that the effects of color combination differed for assimilative and non-assimilative color spreading. They showed that, for assimilative color spreading, interchanging the IC and the OC colors (e.g., changing a lighter orange IC and a darker purple OC combination to a lighter purple IC and a darker orange OC combination) simply changed the induced color that was similar to the IC color. However, if a darker red IC and a lighter magenta OC combination that produced non-assimilative color spreading was changed to a darker magenta IC and a lighter red OC combination, then the non-assimilative color spreading was greatly reduced. These results suggest that the non-assimilative color spreading depends on both luminance and color conditions, just as the assimilative color spreading does. However, for non-assimilative color spreading, the induced color cannot be simply associated with either IC or OC color; the color condition, such as the polarity of chromatic contrast, is apparently critical. Therefore, exploring the color conditions suitable for the non-assimilative color spreading and investigating how the induced color is determined appear to be a good approach to elucidate the properties of the underlying visual mechanisms.

Extending the results of the previous study by Kimura and Kuroki (2014), this study investigated how the induced color in the watercolor configuration was modulated by combinations of the IC and the OC colors, particularly addressing non-assimilative color spreading. The results of analyses in terms of the (L–M) and S cone-opponent responses suggested that the non-assimilative color spreading was mediated mainly by a luminance-dependent S-cone mechanism.

## Experiment 1

In Experiment 1, the IC color was fixed to orange, red, or achromatic, and was combined with the OC color selected from a hue circle centered at the background white color.

### Methods

#### Observers

Five observers (including the second author) participated in Experiment 1. All observers had normal or corrected-to-normal visual acuity and normal color vision as assessed with Ishihara pseudo-isochromatic plates. The four observers other than the author were naïve regarding the purposes of the experiment. All observers who participated in this and subsequent experiments gave informed consent after thorough explanation of the procedures.

#### Apparatus and stimuli

The apparatus for stimulus presentation was described in detail in our previous paper (Kimura and Kuroki, 2014). In brief, the stimuli were generated by a graphic card (VSG 2/5; Cambridge Research Systems) and were displayed on a 21-in. color monitor (GDM F500R; Sony Corp) with a pixel resolution of 1280 × 962 and a frame rate of 80 Hz. The Psychophysics toolbox extensions for Matlab (MathWorks Inc) were used in the colorimetric calculations (Brainard, 1997; Pelli, 1997). A chin and forehead rest was used to maintain a viewing distance of 86 cm. The experiment was run in a dark room.

The two-square configuration was used for this study (Figure 1). The stimulus was composed of outer (3.7°) and inner (2.1°) squares. Color spreading in the corridor area (test area) was investigated. The squares were delineated by sinusoidally shaped double contours (1.9 c/degree, 0.4° peak-to-trough amplitude, and 4.5 min thick). The luminance of a white background was 60 cd/m^2^. Its chromaticity coordinate was *u*′ = 0.1978, *v*′ = 0.4683 in the CIE *u*′*v*′ chromaticity diagram.

To induce strong assimilative and non-assimilative color spreading effects, two luminance conditions were used: the higher and lower IC luminance conditions. In the higher IC luminance condition, the IC luminance was 45 cd/m^2^; the OC luminance was 20 cd/m^2^. In the lower IC luminance condition, the luminance relation was reversed. The IC luminance was 20 cd/m^2^; the OC luminance was 45 cd/m^2^. The higher and the lower IC luminance conditions have been shown to produce assimilative and non-assimilative color spreading, respectively (Pinna et al., 2001; Devinck et al., 2005; Kimura and Kuroki, 2014).

**Figure 2:**
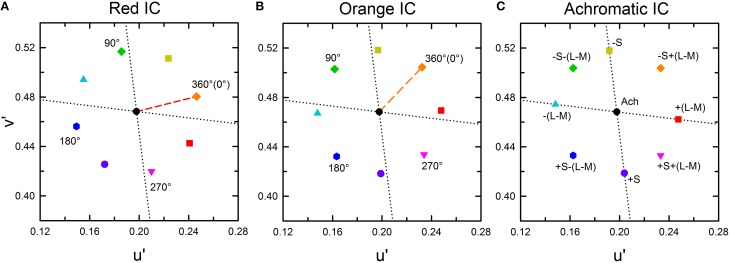
**CIE *u*′*v*′ chromaticity coordinates of the stimulus used for this study. (A)** The red IC, **(B)** orange IC, and **(C)** achromatic IC conditions. In **(A,B)**, the colored dashed line respectively indicates the red and the orange IC color. Numbers in the panel designate the relative angle between the IC and the OC color. The symbol type and color were coded according to the relative angle, but the actual stimulus color corresponding to the same symbol differed [e.g., 360° (0 °) designates red in **(A)** but orange in **(B)**]. The azimuth of the IC color was presented in Table 1. The black circle at the center of the panel designates the background white color (*u*′ = 0.1978, *v*′ = 0.4683). The horizontal and the vertical dotted lines respectively show the directions of the L/(L+M) and the S/(L+M) axes in the cone-excitation diagram of MacLeod and Boynton (1979). In the *u*′*v*′ diagram, the upward and the downward direction respectively correspond to the −S and the +S direction. **(C)** In the achromatic IC condition, the IC color had the same chromaticity coordinate as the background white (designated by the black circle). The label near each symbol represents the relative direction of the OC color in terms of the L/(L+M) and the S/(L+M) axis. For brevity, the L/(L+M) axis was designated as (L–M) and the S/(L+M) axis as S. The same symbol type and color were used to show the results of chromatic cancelation experiments in **Figures 4, 8**.

In each luminance condition, a series of color combinations was tested. All IC and OC colors were located at the Euclidean distance of 0.05 from the white background color in the CIE *u*′*v*′ chromaticity diagram (Figure 2). Three IC colors were used: orange (*u*′ = 0.2297, *v*′ = 0.5068), red (*u*′ = 0.2463, *v*′ = 0.4804), and achromatic (*u*′ = 0.1978, *v*′ = 0.4683). Eight OC colors were combined with each IC color. In the orange and the red IC condition, one OC color was identical to the IC color (i.e., orange and red designated by a dashed line, respectively), with all other OC colors were separated by an angle difference of 45° (Figures 2A,B, and Table 1). In the achromatic IC condition, the OC colors were chosen on the direction of the L/(L+M) axis, the S/(L+M) axis in the cone-excitation diagram proposed by MacLeod and Boynton (1979), or on the intermediate directions between these axes in the CIE *u*′*v*′ chromaticity diagram (Figure 2C, and Table 1). When the IC was achromatic, the induced color was expected to exclusively reflect non-assimilative color spreading from the OC.

**Table 1:** **Color conditions in Experiment 1**.

**Red IC condition**		**Orange IC condition**		**Achromatic IC condition**	
**Relative angle**	**Azimuth**	**Relative angle**	**Azimuth**	**Relative angle**	**Azimuth**
45	59.0	45	91.3	–S	97.0
90	104.0	90	136.2	–S–(L–M)	134.9
135	148.9	135	181.3	–(L–M)	173.0
180	194.0	180	226.3	+S–(L–M)	225.0
225	239.0	225	271.4	+S	277.0
270	284.0	270	316.4	+S+(L–M)	315.1
315	329.1	315	361.4	+(L–M)	353.0
0 (360)	14.0	0 (360)	46.3	–S+(L–M)	45.0

Relative angle of the OC to the IC color and the azimuth of the OC color in the CIE u′v′ chromaticity diagram were listed for different IC conditions (in degrees) (see also Figure 2). The +u′ direction was set to 0 ° and the angle increased in a counterclockwise direction. The azimuth of the red and the orange IC color was, respectively, 14.0 and 46.3 °. For the achromatic IC color, the azimuth was indefinite.

As described above, to analyze the color spreading in terms of cone-opponent responses, the cone-excitation diagram of MacLeod and Boynton (1979) was used. This diagram represents a chromaticity plane of a constant luminance. The luminance is designated by (L+M). The plane is defined by the horizontal axis of L/(L+M) and the vertical axis of S/(L+M), where L, M, and S respectively represent L, M, and S cone excitation. The scaling of the horizontal axis is constrained in that L/(L+M) is the fraction of luminance that is attributable to L cone. However, the scaling of the vertical S/(L+M) axis is unconstrained. For convenience, it was normalized to 1.0 for the background white (*u*′ = 0.1978, *v*′ = 0.4683) in this study. The L/(L+M) and the S/(L+M) line passing through the background white point on the MacLeod and Boynton diagram correspond to the cardinal (L–M) and S axes in the DKL opponent space (Derrington et al., 1984). Consequently, the changes along the L/(L+M) and S/(L+M) axes can be associated with changes in (L–M) and S-(L+M) cone-opponent activities.

#### Procedure

A chromatic cancelation technique was used to quantify color spreading (see Kimura and Kuroki, 2014 for details). Observers adjusted the chromaticity of the test area until it appeared achromatic, whereas the luminance of the test area was held constant at the same luminance as the background. The observer's adjustment was conducted by varying the stimulus along *u*′ and *v*′ axes in the CIE *u*′*v*′ chromaticity diagram. On each trial, the test area chromaticity was preset to a value varied randomly from the background white.

At the beginning of each daily session, the observers dark-adapted for at least 5 min. They then preadapted to the white background for 2 min. Within each session, all stimulus conditions were tested three times in a pseudo-random order. Each session was repeated three times on different days. Therefore, each stimulus condition was tested nine times in all for each observer.

### Cancelation settings for assimilative and non-assimilative color spreading

**Figure 3:**
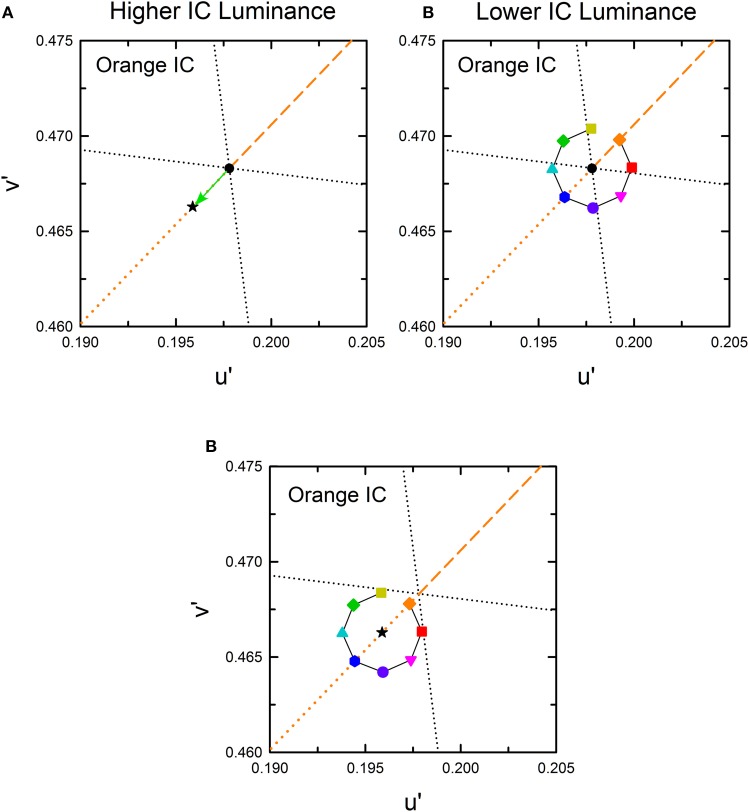
**Expected cancelation results for assimilative and non-assimilative color spreading in the orange IC condition. (A)** Assimilative color spreading. The orange dashed line shows the direction of the IC color, which connects the chromaticity coordinates of the IC color and the background white (designated by the black dot). The orange dotted line shows the complementary color direction of the IC color. Cancelation settings would be on this line, as exemplified by a star symbol if the induced color was the same as the IC color in the higher IC luminance condition. **(B)** Complementary non-assimilative color spreading. The same color as the OC would be necessary to cancel if the induced color was the complementary color of the OC in the lower IC luminance condition. Consequently, the cancelation setting for each OC color would be located in the same direction as the OC color. The symbol type and color correspond to those in Figure 2B. **(C)** An additive color mixture of the assimilative **(A)** and the non-assimilative **(B)** color spreading. Other aspects are the same as those in Figure 2.

According to results obtained from previous studies (Pinna et al., 2001; Devinck et al., 2005, 2006a; Pinna, 2006; Kimura and Kuroki, 2014), different patterns of results are expected in the higher and the lower IC luminance conditions. In the higher IC luminance condition, assimilative color spreading is expected to be predominant and thus the induced color is expected to be similar to the IC color. Consequently, the chromaticity necessary to cancel it would be in the opposite direction to the IC color in a chromaticity diagram, as represented by a black star symbol for the orange IC condition in Figure 3A. In this illustration, the length of the vector (green arrow) connecting the cancelation setting (start symbol) with the background white point (circle) shows the magnitude of color spreading. The direction of the vector indicates the apparent hue of the induced color. All the data symbols representing the cancelation settings for different OC colors are expected to be on the orange dotted line if the results in the higher IC luminance condition are explained completely by chromatic diffusion from the IC. According to Devinck et al. (2006a), different OC colors are able to produce color spreading of different magnitudes but of similar color directions. A combination of the IC and the OC colors that are mutually complementary is expected to induce stronger color spreading. That fact reflects that the induced color is yellower if the cancelation setting deviates to the counterclockwise direction from the orange dotted line. Deviation to the clockwise direction signifies that the induced color is redder.

In the lower IC luminance condition, non-assimilative color spreading is expected to be predominant. According to Pinna (2006), the induced color is expected to be similar to the complementary color of the OC. The cancelation settings for eight OC colors in the orange IC condition would resemble that depicted in Figure 3B if the induced color can be described completely as the complementary color of the OC. The relative position of the colored symbols in the chromaticity diagram is the same as that in Figure 2B because the chromaticity necessary to cancel the complementary color of the OC is expected to be in the same direction as the OC color. Moreover, if the induced color reflects a combination of the chromatic diffusion from the IC and the chromatically opponent induction from the OC, then the result would resemble that depicted in Figure 3C. It represents a combination of the color shifts shown in Figures 3A,B.

### Results and discussion

#### Cancelation settings

**Figure 4:**
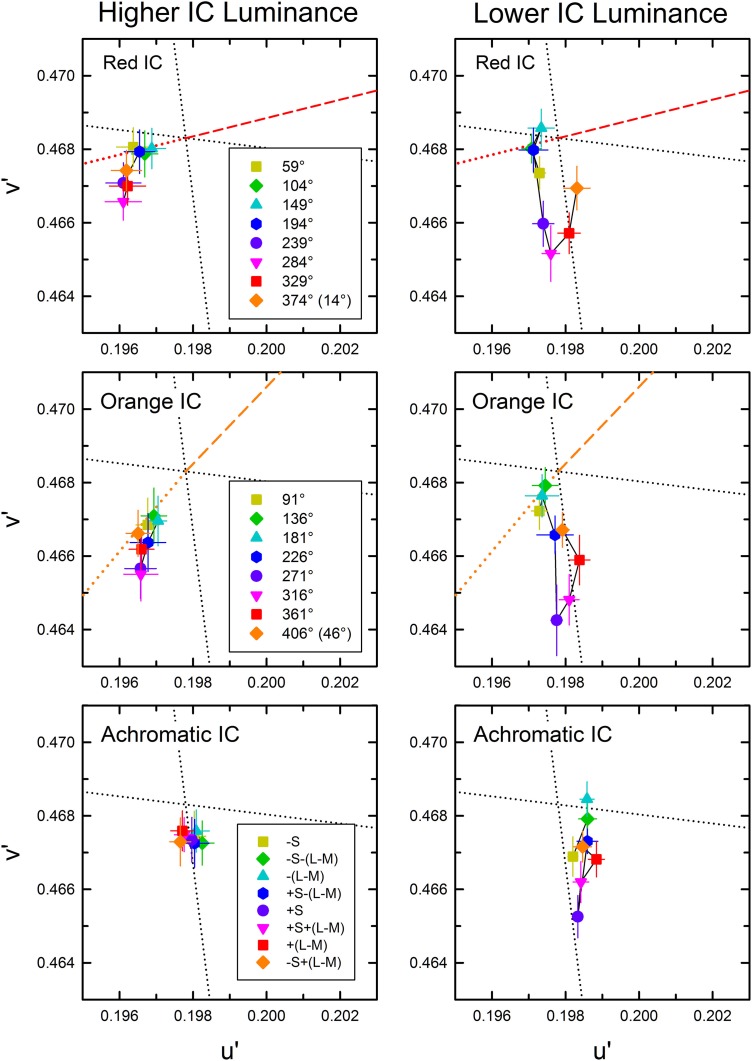
**Chromatic cancelation data of Experiment 1 shown in the CIE *u*′*v*′ chromaticity diagram**. Different symbols designate the mean chromaticity coordinates necessary to cancel the induced color. Left and right panels respectively show results obtained in the higher IC luminance condition and those in the lower IC luminance condition. Top, middle, and bottom panels respectively show results in the red IC, orange IC, and achromatic IC conditions. Correspondence between the OC color and colored symbols was presented graphically in Figure 2. Table 1 listed the azimuth of the OC color in the CIE *u*′*v*′ chromaticity diagram and the relative color angle between the IC and the OC. The legends in the figure designate the azimuth of the OC color. Error bars show ±1 SEM across observers. Other aspects are the same as those shown in Figure 2.

The cancelation settings averaged across different observers are shown in the CIE *u*′*v*′ chromaticity diagram (Figure 4). As expected, different patterns of results were found in the higher and the lower IC luminance condition. The effects of the OC color were less in the higher IC color condition (left panels), but they were greater in the lower IC luminance condition (right panels). However, the results also deviated from the expectation. In the higher IC luminance condition (Figure 4, left), when the IC color was red (top left panel) or orange (middle left panel), the cancelation settings are located closely on the dotted line representing the complementary color of the IC. This result indicates that the induced color was mostly determined by the IC color. However, the OC color also appears to affect the induced color. Particularly, the cancelation settings represented by magenta inverted triangles, red squares, and purple circles deviated to the −*v*′ (counterclockwise) direction, which indicates that the induced color was yellower than the IC color. When the IC was achromatic, color spreading from the IC was not expected. However, the cancelation settings were shifted slightly to the +S/(L+M) (downward) direction, which reflects yellow spreading. In the lower IC luminance condition (Figure 4, right), the pattern of the results was somehow similar to that depicted in Figure 3C. However, the color spreading was much stronger in a yellow direction [i.e., +S/(L+M) direction], particularly for the cancelation settings represented by magenta inverted triangles, red squares, and purple circles. In general, these results are consistent with the findings by Kimura and Kuroki (2014), showing that assimilative color spreading was stronger when the IC contrast (the Weber contrast of the IC to the background luminance) was smaller than the OC contrast, whereas non-assimilative yellow spreading was stronger when the IC contrast was greater than the OC contrast. However, more detailed analysis of the induced color is necessary.

#### Analysis of color direction and shift size

**Figure 5:**
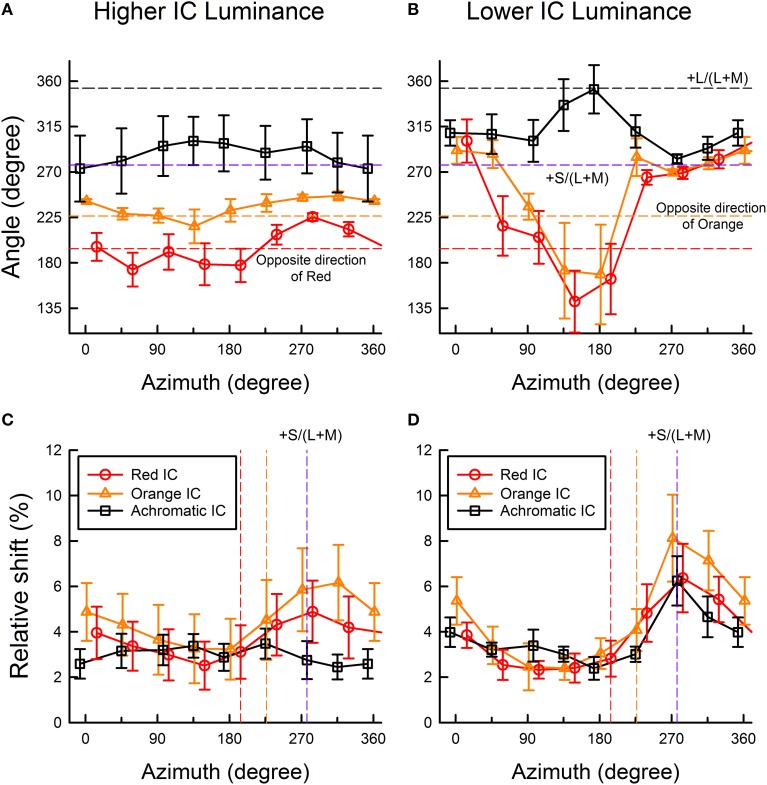
**Color direction and shift size of the cancelation settings as a function of the azimuth of the OC color. (A,C)** show results obtained in the higher IC luminance condition; **(B,D)** show results obtained in the lower IC luminance condition. Upper panels show the results of color direction analysis, whereas lower panels show the results of shift size analysis. The color direction was defined as the angle from the +*u*′ axis to the direction of the mean setting. The direction is opposite to that of the perceived color. The shift size was defined as the vector length (i.e., Euclidean distance) from the background white to the mean setting, expressed in the percentage to the Euclidean distance of the inducing contour colors (0.05). Circle, triangle, and square symbols respectively show results in the red IC, orange IC, and achromatic IC conditions. Error bars show ±1 SEM across observers. In the figures of color direction analysis **(A,B)**, important directions were also shown by horizontal dashed lines [opposite directions of the orange and red colors, +S/(L+M), and +L/(L+M) axis]. In the figures of shift size analysis **(C,D)**, the opposite directions of the orange and red colors, and the +S/(L+M) axis were shown (from left to right) as colored vertical dashed lines.

To quantify the changes in the induced color further, the color direction and shift size of each observer's cancelation settings were calculated for a *u*′*v*′ chromaticity diagram and were then averaged across observers (Figure 5) (See Kimura and Kuroki, 2014 for details of the analysis). The color direction was defined as the angle from the +*u*′ axis to the direction of the mean cancelation setting. The color direction would be 194.0° in the red IC condition and 226.3° in the orange IC condition if the induced color had the same hue as the IC color. The magnitude of color spreading was quantified with the shift size defined as the distance to the mean cancelation setting from the background white point, expressed in the percentage to the Euclidean distance of the inducing contour colors (0.05). The result of the color direction analysis in the higher IC luminance condition (Figure 5A) showed that the color directions of the cancelation settings were similar to those of the IC color in the red IC and the orange IC condition (circle and triangle symbols, respectively), which denotes assimilative color spreading. A One-Way repeated-measures ANOVA showed a significant main effect of the OC color in the red IC and the orange IC condition [*F*_(7, 28)_ = 4.381, *p* = 0.0022 and *F*_(7, 28)_ = 2.611, *p* = 0.0330, respectively]. Multiple comparison tests (Ryan, α = 0.05) showed that the angle of the color direction was larger when the azimuth of the OC color was 284.0° than when it was 59.0, 148.9, or 194.0° in the red IC condition. However, no significant difference was found from multiple comparison tests conducted in the orange IC condition. Consequently, the deviation in the color direction from that of the IC color when the azimuth of the OC color was around 270° (Figure 4A) was statistically significant in the red IC condition.

The result of the shift size analysis (Figure 5C) showed that for these azimuths around 270°, the magnitude of the color spreading was greater in the red IC and the orange IC condition. A One-Way repeated-measures ANOVA showed a significant main effect of the OC color in the red IC and the orange IC condition [*F*_(7, 28)_ = 5.488, *p* = 0.0005 and *F*_(7, 28)_ = 11.319, *p* < 0.0001, respectively]. Multiple comparison tests (Ryan, α = 0.05) showed that the shift size was greater when the azimuth of the OC color was 284.0° than when the azimuth was 104.0, 148.9, or 194.0° in the red IC condition, and that it was greater when the azimuth of the OC was 271.4 or 316.4° than when the azimuth was 46.3, 91.3, 136.2, or 181.3° in the orange IC condition. These results, particularly in the red IC condition, are consistent with the contribution of non-assimilative yellow color spreading. If the complementary color combination of the IC and the OC induced stronger color spreading, then the shift would be larger at the red and the orange vertical line in Figure 5C, which respectively indicate the complementary colors of the red and the orange IC, in the red IC and the orange IC condition. However, the peaks were shifted to a larger azimuth.

In the achromatic IC condition, the color direction was similar to the +S/(L+M) axis (Figure 5A), which indicates yellow spreading. However, the shift was generally small (Figure 5C). A significant main effect of the OC color [*F*_(7, 28)_ = 3.025, *p* = 0.0169] was found from a One-Way repeated-measures ANOVA for the color direction, but no significant difference was found from multiple comparison tests (Ryan, α = 0.05). The ANOVA for the shift size showed that the main effect of the OC color was not significant [*F*_(7, 28)_ = 1.583, *p* = 0.1814].

The result of the color direction analysis in the lower IC luminance condition (Figure 5B) showed much larger and variable changes in apparent hue, particularly when the azimuth of the OC color was 45–225°. The result of the shift size analysis (Figure 5D) showed that within this range of azimuths, the magnitude of the color spreading was small. When color spreading was weak, a small change in the cancelation setting resulted in a large change in color direction. For OC colors producing stronger color spreading (azimuths of around 270°), the color directions of the cancelation settings were very similar to the +S/(L+M) direction (277.0°). This result suggests that stronger yellow non-assimilative color spreading was induced, irrespective of the IC colors, when the azimuth of the OC was about 270° (i.e., the OC color was purple). These tendencies were confirmed using a One-Way repeated-measures ANOVA. The ANOVA for the color direction showed that the main effect of the OC color was significant in all IC color conditions [*F*_(7, 28)_ = 6.033, *p* = 0.0002 for the red IC, *F*_(7, 28)_ = 3.548, *p* = 0.0075 for the orange IC, and *F*_(7, 28)_ = 4.924, *p* = 0.0010 for the achromatic IC condition, respectively]. Multiple comparison tests (Ryan, α = 0.05) showed that the angle of the color direction was smaller when the azimuth of the OC color was 148.9 or 194.0° than when the azimuth was 14.0, or 329.1° in the red IC condition, and that it was larger when the azimuth was 173.0° than when the azimuth was 97.0, 277.0, or 315.1 in the achromatic IC condition. However, in the orange IC condition, none was significant. The ANOVA for the shift size showed that the main effect of the OC color was significant in all IC color conditions [*F*_(7, 28)_ = 5.978, *p* = 0.0003 for the red IC, *F*_(7, 28)_ = 12.147, *p* < 0.0001 for the orange IC, and *F*_(7, 28)_ = 4.142, *p* = 0.0031 for the achromatic IC condition, respectively]. Multiple comparison tests (Ryan, α = 0.05) showed that the shift was larger when the azimuth of the OC color was around 270° and smaller when the azimuth was 45–225° in all IC color conditions.

#### Analysis in terms of cone-opponent activities

**Figure 6:**
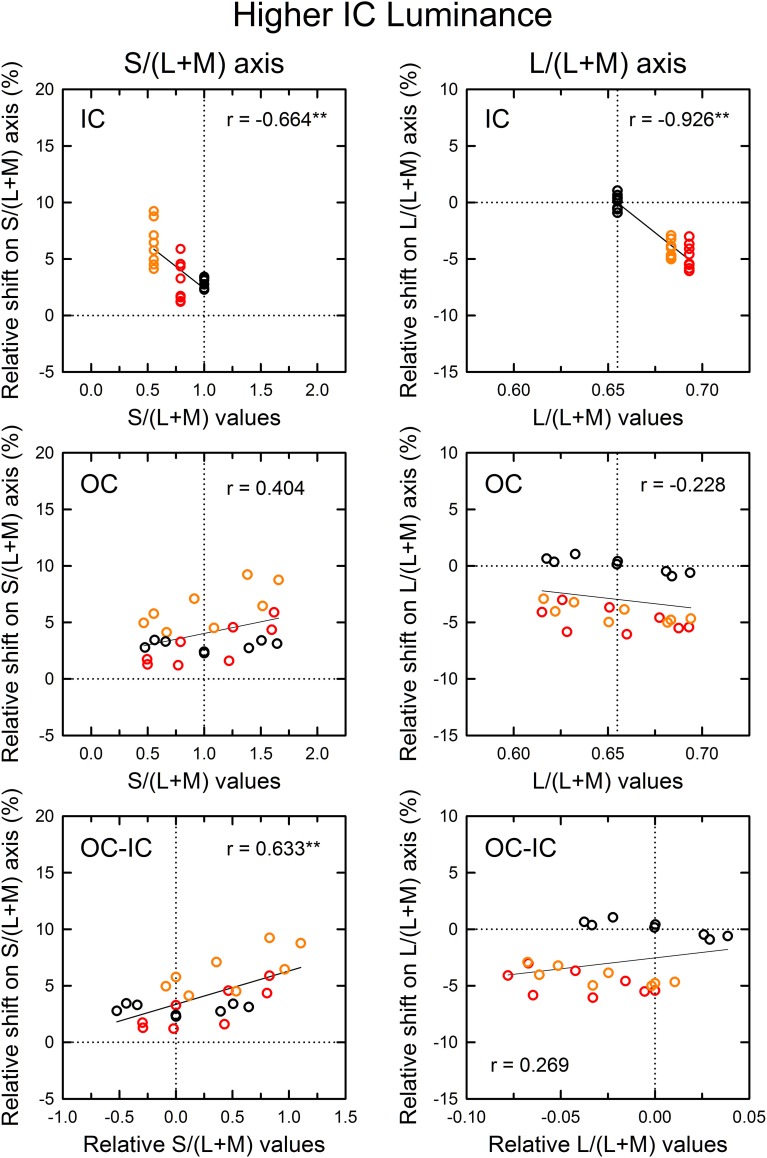
**Results of correlation analysis between the cone excitations caused by inducing contours and the magnitude of color spreading in the higher IC luminance condition**. The magnitude of color spreading was quantified as the relative Euclidean distance on the L/(L+M) [or the S/(L+M)] axis to the mean cancelation setting from the background white point, expressed in the percentage to the mean distance of the IC and the OC color on each axis. The sign of the shift (i.e., incremental or decremental change on each axis) was preserved. Positive values respectively indicate yellowish and greenish spreading for the S/(L+M) and the L/(L+M) axis. Negative values respectively indicate bluish and reddish spreading for the S/(L+M) and the L/(L+M) axis. The zero value designated by the horizontal dotted line represents no color spreading on each axis. Left and right panels respectively show results for the S/(L+M) and the L/(L+M) axes. Top, middle, and bottom panels show correlation with the cone excitations produced by the IC color, the OC color, and the difference between the OC and the IC color (OC-IC). Data symbols are coded with the IC color: the results in the red IC, orange IC, and achromatic IC condition were colored respectively with red, orange, and black. The correlation coefficient was also shown with its significance level in each panel (^**^p < 0.01). In the top and the middle panel, the vertical dotted line designates the cone excitation of the background white color. In the bottom panel, it designates the zero value (i.e., no difference in cone excitation).

**Figure 7:**
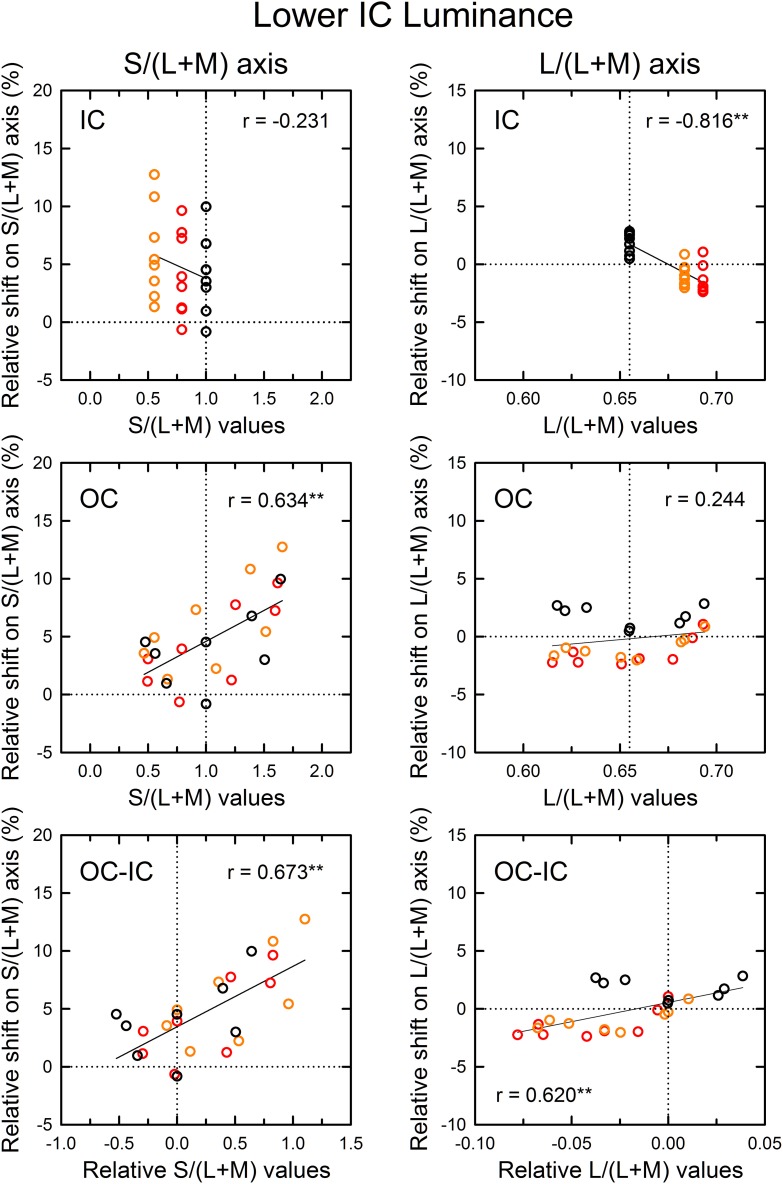
**Results of correlation analysis between the cone excitations caused by inducing contours and the magnitude of color spreading in the lower IC luminance condition**. Left and right panels respectively show results for the S/(L+M) and the L/(L+M) axes. Top, middle, and bottom panels show correlation with the cone excitations produced by the IC color, the OC color, and the difference between the OC and the IC color (OC–IC). Other aspects are the same as those in Figure 6.

To analyze the color spreading in terms of cone-opponent responses, the *u*′ and *v*′ chromaticity coordinates of the cancelation settings as well as the IC and the OC color were transformed into cone excitations along the L/(L+M) and the S/(L+M) axis. For brevity, cone excitations along the L/(L+M) and S/(L+M) axes will be designated respectively as *l* and *s* values. The magnitude of color spreading was quantified as the relative Euclidean distance in terms of the *l* and *s* values to the mean cancelation setting from the background white point, and the sign of the shift (i.e., incremental or decremental change on each axis) was preserved. The shift size was expressed in the percentage to the mean distance of the IC and the OC color on each axis (the mean distance of the inducing contour colors was 0.023–0.025 for the L/(L+M) axis and 0.36–0.39 for the S/(L+M) axis depending on the different IC color conditions). In the analysis, the correlation in cone excitations between the contour color and the induced color was examined (Figures 6, 7) A negative correlation would be shown in the *l* or the *s* values between the IC color and the induced color if (L–M) or S cone-opponent signals diffused from the IC into the test region (chromatic diffusion). However, if (L–M) or S cone-opponent signals by the OC were to induce chromatically opponent signals in the test region (opponent induction), then a positive correlation would be manifested in the *l* or the *s* values between the OC color and the induced color.

The results of the correlation analysis revealed different patterns of results for the L/(L+M) and the S/(L+M) axis (Figures 6, 7). In the higher IC luminance condition (Figure 6), the results for the IC color (top) were similar for the *l* and the *s* values. Both showed highly significant negative correlations [*r* = −0.664, *t*_(22)_ = 4.16, *p* < 0.001 with the *s* values and *r* = −0.926, *t*_(22)_ = 11.52, *p* < 0.001 with the *l* values], which was consistent with the chromatic diffusion. However, the results for the OC color (middle) appear different between the *l* and the *s* values. The results with the *s* values of the OC color showed a positive correlation, which was consistent with the opponent induction, but it was not statistically significant [*r* = 0.404, *t*_(22)_ = 2.07, *p* = 0.0503]. In contrast, the scatter plot of the *l* values showed that the modulation of the OC color only produced small changes in the magnitude of color spreading. The plots were segregated clearly by the IC color. The changes in the magnitude of color spreading were slight for the same IC color conditions. Overall, the results were consistent with the interpretation that, in the higher IC luminance condition, both (L–M) and S cone-opponent mechanisms contributed strongly to the chromatic diffusion, which caused assimilative color spreading.

The patterns of the results in the lower IC luminance condition (Figure 7) were similar to those in the higher IC luminance condition for each cone-excitation axis, but the strength of correlations differed. For the S/(L+M) axis (left), the results for the IC color showed again a negative correlation (top left), but the correlation was not significant [*r* = −0.231, *t*_(22)_ = 1.11, *p* = 0.278]. The slope of the regression line was also smaller (−4.52 in comparison to −7.84 in the higher IC luminance condition). The results for the OC color showed a positive correlation (middle left), which was consistent with the opponent induction. The correlation was highly significant [*r* = 0.634, *t*_(22)_ = 3.84, *p* < 0.001]. The slope of the regression line was greater (5.34 in comparison to 2.05 in the higher IC luminance condition). Consequently, in the lower IC luminance condition, the results suggest that the contribution of an S-cone mechanism to the chromatic diffusion was reduced, although its contribution to the opponent induction was increased.

For the L/(L+M) axis (Figure 7, right), the results for the IC color again showed a highly significant, negative correlation [*r* = −0.816, *t*_(22)_ = 6.62, *p* < 0.001; upper right]. However, the magnitudes of color spreading were close to zero and the regression line became shallower (−86.6 in comparison to −132.7 in the higher IC luminance condition). Consequently, the chromatic diffusion was slight in this condition. The results for the OC color were similar to those in the higher IC luminance condition in that the modulation of the OC color only produced small changes in the magnitude of color spreading for the same IC color conditions (right middle). These results suggest that an (L–M) cone-opponent mechanism mainly contributed to the chromatic diffusion and that its contribution became much smaller in the lower IC luminance condition. Consequently, in the lower IC luminance condition, a significant and large effect was found only in the opponent induction by an S-cone opponent mechanism, which can account for why the color spreading was predominantly yellow in this condition.

Some readers might infer that smaller effects of the OC color found with the *l* values than with the *s* values reflect weaker stimulus modulation on the L/(L+M) axis because of non-uniformity of the *u*′*v*′ chromaticity diagram. The stimulus modulation was equated in the *u*′*v*′ diagram, but its visual effect might not be the same. This non-uniformity can also be reflected in the *l* and the *s* values because the L/(L+M) and the S/(L+M) axes are, respectively, close to the *u*′ and the *v*′ axes (see Figure 4). To investigate the effects of non-uniformity in the *u*′*v*′ diagram, chromatic discrimination thresholds [MacAdam ellipses; MacAdam (1942); Wyszecki and Stiles (1982)] were examined around the white point according to the method used in a study by Devinck et al. (2006a). When the MacAdam ellipse, which has a center closest to the coordinates of our background white color, was placed in the *u*′*v*′ diagram, it was elongated along the *v*′ [or S/(L+M)] axis (see also Figure 6 in Devinck et al., 2006a). To equate the scaling of the two axes, the *u*′ axis must be expanded by 1.72. However, this scaling is much smaller than that necessary to compensate the asymmetry observed in the shift size of the color spreading on different axes in the lower IC luminance condition (2.83 for the red IC and 3.27 for the orange IC condition) (Figure 4, right). Therefore, the difference in the strength of the stimulus modulation on different directions in the chromaticity diagram cannot fully account for the smaller effects of the OC color found with the *l* values.

As demonstrated in previous studies (Devinck et al., 2006a), if the magnitude of assimilative color spreading became greater with a difference between the IC and OC colors and reached its maximum when the two colors were complementary, then the correlation between the OC–IC difference and the magnitude of color spreading are expected to be positive. This prediction was also tested in the higher IC luminance condition (Figure 6, bottom). Results showed that it held true for the *s* values; a highly significant positive correlation was found [*r* = 0.633, *t*_(22)_ = 3.84, *p* < 0.001; bottom left]. However, it did not hold for the *l* values [*r* = 0.269, *t*_(22)_ = 1.31, *p* = 0.204; bottom right]. In the lower IC luminance condition (Figure 7), a significant positive correlation with the *s* values was also found [*r* = 0.673, *t*_(22)_ = 4.27, *p* < 0.001; bottom left]. The correlation with the *l* values was also significant [*r* = 0.620, *t*_(22)_ = 3.71, *p* = 0.0012; bottom right], but for the same IC color conditions, changes in the magnitude of color spreading were small, as in the higher IC luminance condition.

In the correlation analysis with the IC color in the higher IC luminance condition (Figure 6), the results with the *s* values showed small shifts under the achromatic IC condition (black circles in the top left panel; see also Figure 4, lower left). If the induced color was accounted for exclusively by the chromatic diffusion, then the shift size should be zero, as found with the *l* values (black circles in the top right panel in Figure 6). This result suggested the contribution of the opponent induction from the OC. However, the correlation analysis with the *s* values of the OC showed that the magnitude of color spreading did not change much with the *s* values of the OC color (black circles in the middle left panel). Therefore, the correlation was extremely small [*r* = 0.012, *t*_(6)_ = 0.03, *p* = 0.978]. Particularly in this condition, the observers often claimed that cancelation of the induced color was more difficult than in the other conditions. They reported that the adjustment of chromaticity coordinates alone could not cancel the induced color completely. Therefore, they stopped the adjustment when the test region appeared least colored. For complete cancelation, additional brightness adjustment might have been necessary in this condition.

In summary, although our analysis deals only with correlation, the present results are consistent with the interpretation that an S cone-opponent mechanism contributed both to the chromatic diffusion and opponent induction, whereas an (L–M) cone-opponent mechanism mainly contributed to the chromatic diffusion. Both the chromatic diffusion and the opponent induction changed in magnitude with luminance conditions. Their combined effects can account for the apparent hue and the magnitude of assimilative and non-assimilative color spreading.

## Experiment 2

The objective of Experiment 2 was to investigate properties of the S-cone mediated color-spreading (i.e., chromatic diffusion and opponent induction) further by choosing the IC color on the S/(L+M) axis and combining with various OC colors. Particularly, Experiment 2 investigated the effect of hue reversal between the IC and the OC on color spreading. Kimura and Kuroki (2014) showed that non-assimilative yellow color spreading was specific to color combinations. For example, a combination of a dark red IC and a light magenta OC can induce non-assimilative yellow spreading, but with a reversed hue combination, i.e., a combination of a dark magenta IC and a light red OC, color spreading was much reduced. Because the S-cone excitation of the IC color was changed only in the −S direction in Experiment 1 (see top left panels in Figures 6, 7), an incremental S-cone excitation change was also used for the IC color. Then the effects of hue reversal were also investigated.

### Methods

#### Observers

Three observers participated in Experiment 2. Two of the three observers (including the second author) also participated in Experiment 1. A new observer had normal visual acuity and normal color vision, as assessed with Ishihara pseudo-isochromatic plates. Two observers other than the author were naïve regarding the purposes of the experiment.

#### Stimuli and procedure

The same two-square configuration as that used in Experiment 1 was also used in Experiment 2. All three IC colors, +S, −S, and achromatic, were chosen on the S/(L+M) direction and were the same as those used for the OC or IC colors in the achromatic IC condition in Experiment 1 (Figure 2C). Eight different OC colors were also chosen in the same way as in the achromatic IC condition. To induce strong non-assimilative color spreading, the luminance condition was the same as the lower IC luminance condition in Experiment 1: the IC luminance was 20 cd/m^2^; the OC luminance was 45 cd/m^2^.

At the beginning of each daily session, the observers dark-adapted for at least 5 min and then preadapted to the white background for 2 min. Within each session, all stimulus conditions were tested five times in a pseudo-random order. Each session was repeated twice on different days. Therefore, each stimulus condition was tested 10 times in all for each observer. All other aspects of the methods were the same as those in Experiment 1.

### Results and discussion

**Figure 8:**
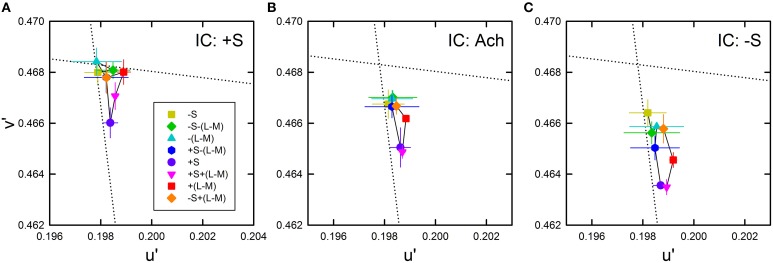
**Chromatic cancelation data of Experiment 2 shown in the CIE *u*′*v*′ chromaticity diagram**. Results obtained when the IC color was +S **(A)**, achromatic **(B)**, and −S **(C)**. Different symbols show the mean chromaticity coordinates necessary to cancel the induced color. The combination of symbol type and color designates the OC color (see also Figure 2C and Table 1). Error bars show ±1 SEM across observers. The IC colors were chosen on the S/(L+M) axis (vertical dotted line). They are therefore not shown graphically in the figure. Other aspects are the same as those shown in Figure 4.

The average cancelation settings were shown in the CIE *u*′*v*′ chromaticity diagram (Figure 8). Regardless of the IC color, almost all cancelation settings were located below the background white point and close to the +S/(L+M) direction, which indicates yellow color spreading. The different IC colors appear to change the magnitude of the yellow spreading and the IC of −S color produced the strongest spreading. It is important to notice that even when the IC color was +S (i.e., purple), the induced color was yellow. This means that non-assimilative yellow spreading was stronger than the typical assimilative color spreading in the present luminance condition where the IC contrast was greater than the OC contrast. Moreover, the yellow spreading was generally stronger when the OC color was +S (purple circle in each panel in Figure 8). It is noteworthy that a strong color spreading was induced when both the IC and OC colors were +S and thus there was no chromatic contrast between the IC and the OC (Figure 8A). Consequently, chromatic contrast between the double contours is not the necessary condition for non-assimilative color spreading. Similarly, when both the IC and OC colors were −S (yellow square in Figure 8C), color spreading of moderate size was induced. Finally, the effect of hue reversal between the IC and the OC color on color spreading was confirmed: strong color spreading was induced when the IC color was −S and the OC color was +S (purple circle in Figure 8C), whereas spreading was extremely small when the IC color was +S and the OC color was −S (yellow square in Figure 8A).

**Figure 9:**
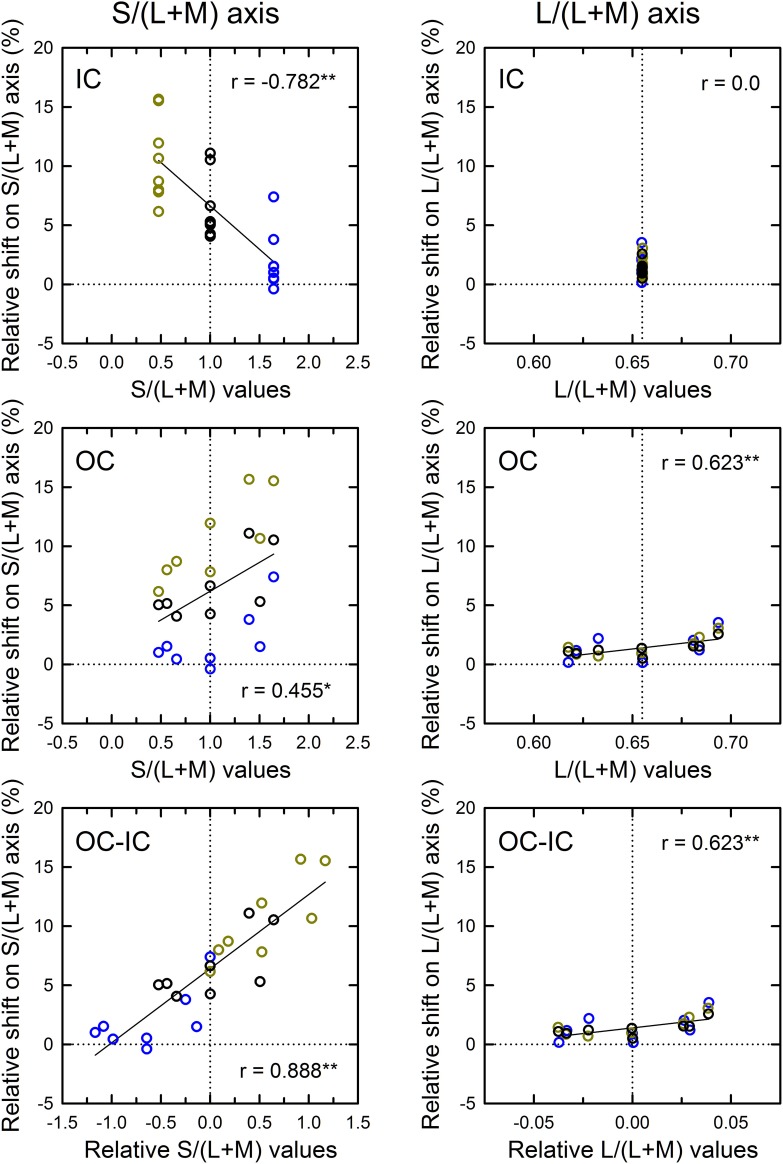
**Results of correlation analysis between the cone excitations caused by inducing contours and the magnitude of color spreading in Experiment 2**. Left and right panels respectively show results for the S/(L+M) and the L/(L+M) axes. Top, middle, and bottom panels respectively show correlation with the cone excitations produced by the IC color, the OC color, and the difference between the OC and the IC color (OC-IC) (^**^p < 0.01, ^*^p < 0.05). Data symbols are coded with the IC color. The results in the −S, achromatic, and +S IC condition were colored respectively with yellow, black, and blue. Other aspects are the same as those in Figure 6.

The correlation in cone excitations between the contour color and the induced color was also examined (Figure 9). Results for the S/(L+M) axis (Figure 9, left) showed that correlation with the *s* values of the IC was negative and highly significant [*r* = −0.782, *t*_(22)_ = 5.88, *p* < 0.001; top left]. Greater modulation of the *s* values in Experiment 2 than in Experiment 1 made the negative correlation significant (see also the top left panel in Figure 7). Correlation with the OC color was also significant [*r* = 0.455, *t*_(22)_ = 2.40, *p* = 0.025; middle left]. These results are consistent with the contributions of both the chromatic diffusion and the opponent induction by an S cone-opponent mechanism. Combining the effects of the IC and OC colors as the OC–IC difference in the *s* values caused a very high positive correlation [*r* = 0.888, *t*_(22)_ = 9.06, *p* < 0.001; bottom left]. Moreover, the result showed clearly that even when the difference in the *s* values between the IC and the OC color was zero (i.e., S-cone contrast was zero; the vertical dotted line in the bottom left panel), the S-cone mediated color-spreading was induced. Consequently, S-cone contrast between the inducing double contours was not necessary for the S-cone mediated color-spreading. This color spreading cannot be explained by the chromatic diffusion because some data were obtained when the IC color was −S or achromatic (yellow and black circles on the vertical dotted line in the bottom left panel). In the S-cone mediated color-spreading, the opponent induction [−S+(L+M) type] was stronger than the chromatic diffusion, which can account for the effect of hue reversal between the IC and the OC on color spreading (Kimura and Kuroki, 2014).

The results for the L/(L+M) axis (Figure 9, right) showed again that the modulation of the OC color produced only small changes in the magnitude of color spreading (middle right), although the correlation was significant [*r* = 0.623, *t*_(22)_ = 3.73, *p* = 0.001].

## General discussion

This study investigated how the induced color in the watercolor configuration was determined, particularly addressing non-assimilative color spreading. The present results are consistent with the interpretation that the induced color can be accounted for by a combination of diffusion of cone-opponent signals from the IC (chromatic diffusion) and chromatically opponent induction from the OC (opponent induction). Moreover, the chromatic diffusion and the opponent induction are mediated differentially by (L–M) and S-cone opponent mechanisms. The chromatic diffusion was mediated by both (L–M) and S-cone opponent mechanisms. In contrast, the opponent induction was mediated mainly by an S-cone opponent mechanism [particularly, −S+(L+M) type] and the contribution of an (L–M) mechanism was very small (Figures 6, 7, 9). Therefore, the influence of the S-cone mechanism was dissociable from that of the (L–M) mechanism. To elucidate to what degree and how the (L–M) mechanism contributes to the opponent induction, further studies using much greater modulation of (L–M) contrast are necessary.

Moreover, these differential contributions of cone-opponent mechanisms depended on the luminance conditions. Chromatic diffusion by the (L–M) and the S-cone mechanism increased when the Weber contrast of the IC to the background luminance (IC contrast) was smaller in size than the OC contrast (higher IC luminance condition, Figure 6). The opponent induction by the S-cone mechanism was weak. The combination of these effects accounted for the color spreading in the higher IC luminance condition; mostly assimilative color spreading, but additional yellow spreading when the OC produced greater S-cone excitations. This yellow spreading is also associated with the results that color spreading deviated from the color direction of the IC color when the azimuth of the OC color was around 270° (Figure 4, left). When the luminance condition was opposite and the IC contrast was greater than the OC contrast (lower IC luminance condition, Figure 7), the opponent induction by the S-cone mechanism increased. In contrast, the chromatic diffusion by the S-cone mechanism became smaller in this luminance condition, although it can contribute significantly to color spreading when the S-cone modulation in the IC was large (Figure 9, top left). Consequently, the color spreading in the lower IC luminance condition mostly reflects the S-cone mediated opponent induction. The induced color was yellow, because it was mostly −S+(L+M) type, i.e., the −S (yellowish) induction was stronger than the +S (bluish) induction. The strong yellowish opponent induction can obscure the S-cone mediated chromatic diffusion even when the chromatic diffusion produced the color spreading of the +S (bluish) direction (Figure 8A). Overall, the dependency of color spreading on luminance conditions were consistent with previous findings (Pinna et al., 2001; Devinck et al., 2005; Cao et al., 2011; Kimura and Kuroki, 2014) and suggested that the watercolor effect is mediated by luminance-dependent chromatic mechanisms (Devinck et al., 2006a).

The present results also demonstrate that the magnitude of color spreading was highly correlated with the OC–IC difference in S-cone excitations (bottom left panels in Figures 6, 7, 9). As discussed earlier, this finding is consistent with the interpretation that the S-cone mediated color spreading is accounted for by a combination of the chromatic diffusion and the opponent induction. However, another possibility can be conceived: the S-cone mediated color spreading is accounted for by a single spreading effect depending on S-cone contrast between the IC and the OC. This possibility appears plausible in view of earlier findings. Devinck et al. (2006b) showed that the magnitude of the watercolor effect was similar for two spatial profiles having sharp or smooth transitions between the two contours when the contours were narrow. Moreover, several previous studies examined the relation between the watercolor effect and the Craik–O'Brien–Cornsweet effect (e.g., Pinna et al., 2001; Cao et al., 2011). However, some of the present results seem contradictory to this possibility. If the spreading depended on S-cone contrast between the IC and the OC, it would disappear when the IC and the OC color were the same. However, the results of Experiment 2 showed that this is not the case (Figures 8A,C). Additionally, the analysis of cone excitations showed that S-cone mediated color spreading can be found even when S-cone contrast was zero (Figure 9, lower left). In retrospect, similar results were also found in Experiment 1 (Figure 4, right). When the relative angle between the IC and the OC color was zero (orange diamond symbol), yellow spreading of moderate size was induced. Consequently, the present results suggest that the combination hypothesis is more likely.

In contrast to S-cone excitations, the effects of the OC–IC difference along the L/(L+M) axis on the magnitude of color spreading was not strong in both higher and lower IC luminance conditions, mainly because of small effects of the OC color modulation. Therefore, the magnitude of color spreading is not maximal when the IC and the OC color are complementary. This finding is not consistent with previous findings (Devinck et al., 2006a). However, we have no plausible explanation for this inconsistency. We wondered that some differences in stimulus conditions between these studies, notably the duration of the stimulus presentation, could account for the difference: Devinck et al. (2006a) used a stimulus sequence of 2-s presentation and 2-s interstimulus interval to reduce visual adaptation to the stimulus patterns. In an additional experiment, we repeated the present Experiment 1 using the same stimulus sequence. However, the results were almost identical to those in Experiment 1. Therefore, a difference in visual adaptation is not a likely cause of the inconsistency. Additionally, small contributions of an (L–M) cone-opponent mechanism to the color spreading in the lower IC luminance condition led to the results that the color-spreading was predominantly yellow, irrespective of the OC color. This finding is inconsistent with previous results reported by Pinna (2006) showing complementary color spreading with black IC and chromatic OC combinations. This inconsistency might be accounted for by differences in saturation of the inducing contours (see also Kimura and Kuroki, 2014). Pinna (2006) used much more saturated OC colors than those used in the present study. Those saturated colors might have led to significant opponent induction caused by an (L–M) cone-opponent mechanism. In other words, contrast gain of the (L–M) mediated opponent induction might be smaller than that of the S-cone mediated induction. Moreover, Pinna (2006) reported that a black IC and a yellow OC combination can produce chromatically opponent blue spreading. A +S type of opponent induction, which is opposite to the −S type induction found in this study, was apparently also observable with highly saturated stimuli.

Finally, it is noteworthy that all the present results are consistent with the theory that color spreading in the watercolor configuration was mediated by luminance-dependent (L–M) and S cone-opponent mechanisms. The magnitude and the apparent hue of the color spreading were affected strongly by the luminance contrast between the double contours. These results suggest the contribution of cortical chromatic mechanisms to color spreading in the watercolor effect. Physiological studies showed that luminance and S-cone signals are largely segregated in subcortical neurons (e.g., Solomon and Lennie, 2007), but many neurons in V1 respond to both luminance and chromatic signals (Johnson et al., 2001, 2004; Xiao, 2014). Moreover, although neurons with S-cone spatial antagonism have not been found in the retina (Dacey, 2000), some evidence exists that V1 neurons exhibit the antagonism (Conway, 2001; Solomon et al., 2004). In fact, a receptive field model with S-cone center-surround antagonism has been proposed to explain chromatic induction from S-cone patterns composed of fine lines (Monnier and Shevell, 2004; Shevell and Monnier, 2005). Cortical neurons with spatial antagonism in luminance and chromatic signals might be responsible for color spreading in the watercolor effect.

From this physiological viewpoint, the present findings provided several constraints on possible visual mechanisms. First, color spreading depends on a combination of luminance and chromatic contrasts between the double contours, but the polarity of the contrasts is crucially important for determining the magnitude and the apparent hue of the color spreading. Similar response dependence on contrast polarity of boundaries has been found in cortical neurons coding border ownership (Zhou et al., 2000; von der Heydt et al., 2003). However, the dependence on specific combinations of contrast polarity must be explored. Secondly, the finding that opponent induction was mediated mainly by a −S inducing mechanism, at least in the present stimulus condition, is consistent with the enhancement of S-off signals in V1 (Xiao, 2014). However, its differential contribution is unexpected. Thirdly, the contributions of the S cone-opponent mechanism to color spreading can be largely separable from those of the (L–M) mechanism (Figures 7, 9). This finding might require modification of the cortical model of color spreading because physiological studies have showed that S-cone sensitive neurons in V1 also receive robust (L–M) cone-opponent inputs (Conway, 2001; Solomon and Lennie, 2005; Xiao, 2014).

### Conflict of interest statement

The authors declare that the research was conducted in the absence of any commercial or financial relationships that could be construed as a potential conflict of interest.
